# Supplementary material for: Achievement of the low-density lipoprotein cholesterol goal among patients with dyslipidemia in South Korea
Source: PLoS One. 2020 Jan 30;15(1):e0228472. doi: 10.1371/journal.pone.0228472 (PMC6992159; doi:10.1371/journal.pone.0228472)
Supplement: S1 Table — (DOCX) [file pone.0228472.s001.docx]

S1 Table. Diagnosis and procedure codes to identify very high- and high-risk groups

| **Risk groups** | **Items** | **ICD-10/procedure codes** |
| --- | --- | --- |
| Very high risk | Angina | I20 |
|  | Myocardial infarction | I21 |
|  | Ischemic stroke | I63 and hospitalization |
|  | Transient ischemic attack | G45.0, G45.1, G45.2, G45.3, G45.8, G45.9, G46.0, G46.1, G46.2 |
|  | Peripheral artery disease | I65, I66, I70, I73.9, I74 |
|  | Coronary revascularization  (procedure codes) | HA670, HA680, HA681, HA682, M6551, M6552, M6561, M6562, M6563, M6564, M6571, M6572, M6634, O1641, O1642, O1647, O1830, OA641, OA642, OA647, M6620, M6633  O2053, O2057, OA631, OA634, OA635, OB631, OB634, OB635 |
|  | Peripheral revascularization  (procedure codes) | M6593, M6594, M6595, M6596, M6597, M6599, M6601, M6602, M6603, M6604, M6605, M6611, M6612, M6613, M6632, O0161, O0162, O0163, O0164, O0165, O0166, O0167, O0168, O0169, O0170, O0171, O0226, O0227, O1643, O1644, O1645, O1646, O1950, O2054, O2055, O2056, O2058, O2059, O2064, O2065, O2066, O2067, O2068, OA632, OA633, OA636, OA637, OA638, OA639, OB632, OB633, OB636, OB637, OB638, OB639 |
| High risk | Abdominal aneurysm | I71.3, I71.4 |
|  | Diabetes mellitus | E10, E11, E13, E14 |
| ICD-10, International Classification of Diseases, Tenth Revision. | | |
