# Supplementary material for: Achievement of the low-density lipoprotein cholesterol goal among patients with dyslipidemia in South Korea
Source: PLoS One. 2020 Jan 30;15(1):e0228472. doi: 10.1371/journal.pone.0228472 (PMC6992159; doi:10.1371/journal.pone.0228472)
Supplement: S2 Table — (DOCX) [file pone.0228472.s002.docx]

S2 Table. Diagnosis codes to define cardiovascular events

| **Cardiovascular events** | **ICD-10 codes** |
| --- | --- |
| Acute coronary syndrome | I20.0, I21 |
| Ischemic stroke | I63 and hospitalization |
| Peripheral artery disease | I65, I66, I70, I73.9, I74 |
| Cardiovascular death^a^ | I20, I21, I63, I65, I66, I70, I73, I74 |
| ICD-10, International Classification of Diseases, Tenth Revision.  ^a^Cardiovascular death was defined if a patient had ICD-10 codes of cardiovascular events as the causes of death. | |
