# Supplementary material for: Achievement of the low-density lipoprotein cholesterol goal among patients with dyslipidemia in South Korea
Source: PLoS One. 2020 Jan 30;15(1):e0228472. doi: 10.1371/journal.pone.0228472 (PMC6992159; doi:10.1371/journal.pone.0228472)
Supplement: S3 Table — (DOCX) [file pone.0228472.s003.docx]

S3 Table. LDL-C goal achievement rates based on the 2015 Korean guidelines and LDL-C distributions categorized by the use of lipid-modifying treatment

| **Category** | **LDL-C goal achievement rate**  **(%)** | **Average LDL-C**  **(mg/dL)** | | **Recommended LDL-C goal**  **(mg/dL)** |
| --- | --- | --- | --- | --- |
|  |  | **Achievers** | **Non-achievers** |  |
| All patients |  |  |  |  |
| No LMT | 40.2 | 103.7 | 139.9 |  |
| Adherent (statin) | 58.7 | 76.6 | 105.0 |  |
| Adherent (non-statin) | 39.1 | 90.0 | 126.8 |  |
| Non-adherent | 48.1 | 88.6 | 131.7 |  |
| Very high-risk group |  |  |  |  |
| No LMT | 5.5 | 54.1 | 126.4 | 70 |
| Adherent (statin) | 29.4 | 55.3 | 98.8 | 70 |
| Adherent (non-statin) | 10.1 | 50.3 | 120.4 | 70 |
| Non-adherent | 17.1 | 54.5 | 116.5 | 70 |
| High-risk group |  |  |  |  |
| No LMT | 28.7 | 78.9 | 135.9 | 100 |
| Adherent (statin) | 73.8 | 72.5 | 121.2 | 100 |
| Adherent (non-statin) | 39.1 | 77.6 | 126.2 | 100 |
| Non-adherent | 48.6 | 73.7 | 136.1 | 100 |
| Moderate-risk group |  |  |  |  |
| No LMT | 54.7 | 99.7 | 158.6 | 130 |
| Adherent (statin) | 90.4 | 85.7 | 151.2 | 130 |
| Adherent (non-statin) | 67.7 | 96.5 | 153.5 | 130 |
| Non-adherent | 67.7 | 91.9 | 162.5 | 130 |
| Low-risk group |  |  |  |  |
| No LMT | 77.4 | 118.1 | 183.7 | 160 |
| Adherent (statin) | 97.2 | 94.5 | 193.4 | 160 |
| Adherent (non-statin) | 92.2 | 114.8 | 177.5 | 160 |
| Non-adherent | 81.9 | 106.6 | 187.4 | 160 |
| LDL-C, low-density lipoprotein cholesterol; LMT, lipid-modifying treatment. | | | | |
