# Supplementary figures and images for: Achievement of the low-density lipoprotein cholesterol goal among patients with dyslipidemia in South Korea
Source: PLoS One. 2020 Jan 30;15(1):e0228472. doi: 10.1371/journal.pone.0228472 (PMC6992159; doi:10.1371/journal.pone.0228472)

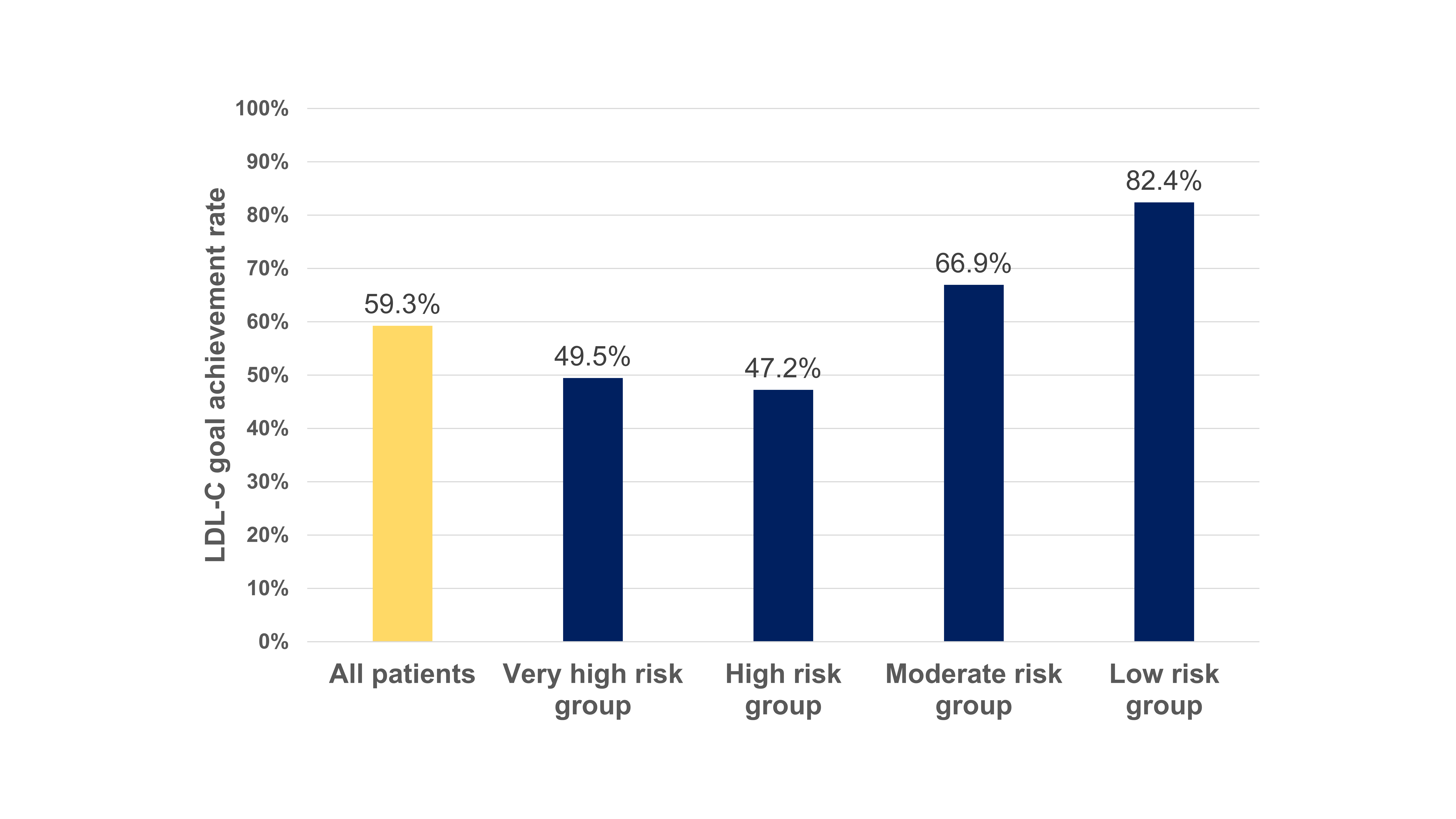

Supplement: S1 Fig — (TIF) [file pone.0228472.s004.tif]
